# Risk Factors for Septic Arthritis After Anterior Cruciate Ligament Reconstruction: A Nationwide Analysis of 26,014 ACL Reconstructions

**DOI:** 10.1177/0363546521993812

**Published:** 2021-03-25

**Authors:** Jesper Kraus Schmitz, Viktor Lindgren, Gunnar Edman, Per-Mats Janarv, Magnus Forssblad, Anders Stålman

**Affiliations:** † Stockholm Sports Trauma Research Center, Department of Molecular Medicine and Surgery, Karolinska Institutet, Stockholm, Sweden; ‡ Department of Orthopaedics, Skåne University Hospital, Malmö, Sweden; § Department of Molecular Medicine and Surgery, Karolinska Institutet, Stockholm, Sweden; ‖ Department of Orthopaedics, Karolinska University Hospital, Stockholm, Sweden; ¶ Research and Development, Norrtälje Hospital, Tiohundra AB, Norrtälje, Sweden; # Department of Clinical Sciences, Danderyd Hospital, Karolinska Institutet, Stockholm, Sweden; ** Capio Artro Clinic, Stockholm, Sweden; Investigation performed at Stockholm Sports Trauma Research Center, Stockholm, Sweden

**Keywords:** ACL reconstruction, infection, septic arthritis, incidence, risk factors

## Abstract

**Background:**

Septic arthritis (SA) after anterior cruciate ligament reconstruction (ACLR) is a rare yet severe complication. The samples in previous studies have been small and without nationwide coverage, making analysis uncertain with a risk of bias. Conclusions to recommend preventive measures are therefore difficult to draw, and it has not been possible to perform a comprehensive risk factor analysis.

**Purpose:**

To study the incidence of SA after ACLR in a large, nationwide population and to study the risk factors for SA after ACLR.

**Study Design:**

Case-control study; Level of evidence, 3.

**Methods:**

All ACLRs, primary and revision, in the Swedish Knee Ligament Registry between 2006 and 2013 were linked with data from the Swedish National Board of Health and Welfare. The incidence of SA events was determined using entries from the day of surgery until 90 days postoperatively based on diagnosis codes and the prescription of antibiotics. All events of SA were verified via a review of medical records. Risk factors were analyzed based on data from the registries. Descriptive statistics were used to describe the findings, while logistic regression analysis was used for the risk analysis.

**Results:**

The cohort consisted of 26,014 primary and revision ACLRs. During the study period, 298 events of SA (1.1%) were identified. The high-volume units (≥500 ACLRs during the study period) had a distribution of SA between 2 and 47 (0.2%-2.9%). Independent risk factors of SA were male sex (OR, 1.65; 95% CI, 1.28-2.13), operating time ≥70 minutes (OR, 1.83; 95% CI, 1.42-2.36), hamstring tendon autograft (OR, 2.23; 95% CI, 1.21-4.08), and clindamycin as perioperative antibiotic prophylaxis (OR, 1.94; 95% CI, 1.10-3.41).

**Conclusion:**

The incidence of SA after ACLR in this nationwide cohort was 1.1%. Male sex, hamstring tendon autografts, and a longer operating time were all independent risk factors for SA. The use of clindamycin as perioperative antibiotic prophylaxis was a risk factor compared with the use of cloxacillin. Some high-volume units had a very low infection rate (0.2%).

Anterior cruciate ligament reconstruction (ACLR) is one of the most common orthopaedic procedures in the United States, with an estimate of 43.5 procedures per 100,000 people; the corresponding number in Sweden is 40.7 procedures per 100,000 people.^22,31^ The population who undergo surgery are usually young and active, with a mean age of 28 years.^31^ One serious complication after ACLR is septic arthritis (SA), which results in prolonged rehabilitation, a poorer outcome, and often the need for repeated surgery.^18^ The incidence of postoperative SA after ACLR in registry-based cohorts has been reported to be between 0.28% and 1.0%.^7,9,36^ Reports from single institutions have shown a slightly higher rate of infection (0.58%-1.8%).^4-6,33^ The different methods of analyzing infections, registry-based cohorts and single institution–based cohorts, both have their advantages and disadvantages. The single-institution setting provides homogeneity in the data, whereas the registry-based studies include data from multiple sites and a possible heterogeneity. The main strength of a multicenter study is its generalizability and how it can describe the occurrence of infection in a population operated on by different surgeons with varying experience and differences in local routines.

To minimize the risk of SA, perioperative antibiotics are routinely used.^10,34^ Previous studies have described diabetes mellitus, smoking, and the use of hamstring autografts as risk factors for postoperative SA.^4,7,9^ However, since the rate of infection is low, the analysis of risk factors is challenging and can be associated with a substantial amount of bias. One way to approach this bias is to include a larger population in a register-based study.

The aim of this study was to establish the incidence of SA after ACLR in a nationwide cohort and perform a comprehensive risk factor analysis for SA.

## Methods

All primary and revision ACLRs registered in the Swedish Knee Ligament Registry (SKLR) between 2006 and 2013 were included. By using personal identity numbers, the data from the SKLR could be cross-matched with the registry data from the National Board of Health and Welfare. All entries between the day of surgery and 90 days after surgery were analyzed.

### Swedish Knee Ligament Registry

The SKLR was established in 2005. It is a nationwide quality registry of ACLRs and covers >90% of all ACLRs. The registry consists of 2 parts: reports and perioperative observations by the surgeon (associated injuries, type of graft, use of antibiotics, type of graft fixation) and patient self-reported data (body mass index [BMI], smoking).^31^

### Registries From the National Board of Health and Welfare

The National Patient Registry (NPR) was started in 1964 and, since 2001, has included all in- and outpatient care in Sweden excluding primary health care. It consists of patient data (personal identity number, sex, age, place of residence), geographical data (hospital), administrative data (date of admission/discharge), and medical data (primary and secondary diagnoses, procedures). Diagnoses are coded according to the International Classification of Diseases (ICD), 10th Revision (ICD-10).^19,30^

The Swedish Prescribed Drugs Registry (SPDR) was started in July 2005 and contains a record of all dispensed drugs. It consists of patient data (personal identity number, sex, age, place of residence) and drug data (name, dose, dispensed amount).^37^

The Causes of Death Registry was started in 1961 and includes all deaths and causes of death in Sweden.^29^

### Incidence Analysis

**Figure 1.**
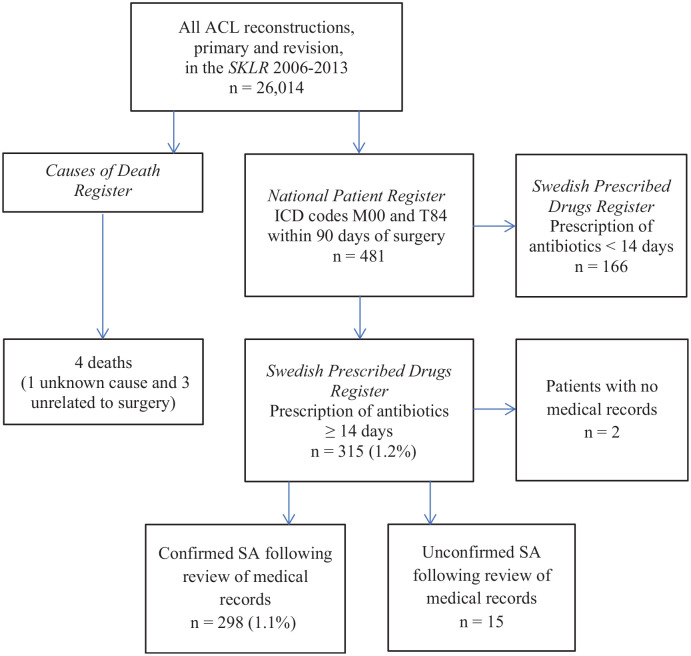
Flowchart of patient selection. ACL, anterior cruciate ligament; ICD, International Classification of Diseases; M00, pyogenic arthritis; SA, septic arthritis; SKLR, Swedish Knee Ligament Registry; T84, complications of internal orthopaedic prosthetic devices, implants, and grafts.

SA was defined according to a combination of specific ICD-10 codes (M00, pyogenic arthritis, and T84, complications of internal orthopaedic prosthetic devices, implants, and grafts) and the prescription of antibiotics in the SPDR for at least 14 days (Appendix Table A1, available in the online version of this article). This method was used to ensure that the event of SA was significant, did not require prolonged antibiotic therapy, and was not given an incorrect ICD code. To further enhance the analysis, all positive findings in the review of registries were verified via a review of medical records. Patients with a positive culture from joint aspiration were included in the analysis. Patients without a positive culture but with clinical signs of SA, such as temperature ≥38°C, knee effusion, C-reactive protein (CRP) >40, joint fluid with low glucose and increased leukocytes, and treatment of SA, were included in the analysis. Patients without medical records were excluded (n = 2) (Figure 1). Time to SA was defined as the number of days after the day of surgery to the first postoperative visit for health care when SA was diagnosed.

### Risk Factor Analysis

The identified cases in the incidence analysis were analyzed based on the information from the SKLR (sex, BMI, smoking, age at surgery, primary or revision surgery, in- or outpatient surgery, cartilage lesion, meniscal suture, operating time, choice of graft, and perioperative antibiotics). The patients with allograft and other types of graft (quadriceps, direct suture) were small in number and thus excluded from the risk factor analysis. Diabetes mellitus was defined as the multiple prescription of antidiabetic drugs in the SPDR. Perioperative antibiotics were divided into 4 categories (cloxacillin, clindamycin, cefuroxime, and other drugs), and the risk factor analysis included the 2 most used categories. Data of patients with a venous thromboembolism (VTE) were retrieved from a previous study of the same cohort.^17^ Prolonged antibiotic prophylaxis was defined as the prescription of antibiotics on postoperative days 0 to 2.

### Statistical Analysis

Statistical analysis was conducted using SPSS Statistics Version 25.0 (IBM Corp). All the variables were summarized using standard descriptive statistics such as frequency, mean, and SD.

Differences between groups in terms of categorical variables, such as differences in sex between groups with or without SA, were analyzed using Pearson chi-square method. If an expected cell frequency was 5 or less, Fisher exact test was used. Differences between 2 groups in terms of continuous variables were analyzed using the Student *t* test for independent variables, provided that the distributions were not severely skewed. The variables with a statistically significantly difference in the univariate analysis (sex, cartilage lesion, operating time, choice of graft, and type of perioperative antibiotics) were included in a logistic regression analysis of risk factors. BMI was excluded because of a large number of missing values (n = 161 in the SA group, n = 13,253 in the non-SA group). VTE was excluded because of uncertainty regarding causality. The continuous variable of operating time was dichotomized based on the median value of 70.0 minutes (skewness value, 0.95). The relationship between a risk variable and SA was expressed as an odds ratio (OR) with a 95% CI. The level of significance was 5% (2-tailed) in all analyses.

### Ethics

The study was approved by the regional ethics committee at Karolinska Institutet (reference No. 2013/1257-31/3).

## Results

Between 2006 and 2013, a total of 26,014 ACLRs in 24,577 patients, performed at 52 institutions, were registered in the SKLR. There were a total of 1619 (6.2%) revision procedures. The mean age of the patients at the time of surgery was 26.8 years (range, 7-74 years). Three patients died of unrelated causes: 1 of a gunshot wound, 1 from a fall from a height, and 1 from an alcohol-related condition. One patient, a 21-year-old man, died 58 days after surgery of an unknown cause.

The analysis of the NPR and SPDR found 315 patients with suspected SA. After the analysis of medical records, the confirmed events of SA were reduced to 298, corresponding to an incidence of 1.1% (Figure 1). In the 16 high-volume units (≥500 ACLRs during the study period), the distribution of SA was 2 to 47 (0.2%-2.9%). No significant difference in the distribution of SA was found between high-volume and low-volume units (1.1% vs 1.2%; *P* = .273). The overall mean time from surgery to diagnosis was 18.4 days (range, 1-74 days). The distribution of graft choice (hamstring tendon autograft, patellar tendon autograft, allograft, and other graft) was significantly different between the SA group and the non-SA group (*P* = .011); however, the 2 categories of allograft (SA, n = 0; non-SA, n = 142) and other grafts (SA, n = 6; non-SA, n = 275) had small numbers and were excluded from further analysis.

The group with SA had a greater proportion of male patients (68.7% vs 57.2% women; *P* < .001), a greater incidence of postoperative VTE (1.4% vs 0.3%; *P* = .021), and a significantly greater mean BMI (25.4 vs 24.5; *P* = .003). When BMI was analyzed in the categories of normal weight, overweight, and obese, no difference was found between the group with SA and the group without SA (Table 1). The perioperative data demonstrated that the group with SA had a longer mean operating time (82.0 minutes vs 74.5 minutes; *P* < .001) and a greater occurrence of cartilage lesions (34.0% vs 27.1%; *P* = .008). The mean operating time for patients with a cartilage lesion was longer compared with that for patients without a cartilage lesion (79.3 minutes vs 72.9 minutes; *P* < .001). The choice of graft and the choice of perioperative antibiotics were significantly different between the SA group and the non-SA group (Table 2).

**Table 1:** Characteristics of Patients With (n = 291) and Without (n = 25,018) Septic Arthritis^*a*^

	Septic Arthritis		
Variable (Incidence Rate)	Yes	No	*P* Value
Sex (n = 25,309)			
Female (0.8)	91 (31.3)	10,697 (42.8)	<.001
Male (1.4)	200 (68.7)	14,321 (57.2)	
Age at surgery, y, mean (range) (n = 25,309)	27.2 (11-53)	26.8 (7-67)	.556
BMI, mean (SD) (n = 11,895)	25.4 (3.94)	24.5 (3.52)	.003
Normal weight (BMI, <25) (0.9)	71 (54.6)	7425 (63.1)	.132
Overweight (BMI, 25-30) (1.4)	53 (40.8)	3857 (32.8)	
Obese (BMI, >30) (1.2)	6 (4.6)	483 (4.1)	
Smoking (n = 12,169)			
Yes (0.8)	6 (4.5)	713 (5.9)	.504
No (1.1)	126 (95.5)	11,324 (94.1)	
Diabetes mellitus (n = 25,309)			
Yes (1.1)	2 (0.7)	187 (0.7)	>.999
No (1.2)	289 (99.3)	24,831 (99.3)	
VTE (n = 25,309)			
Yes (4.4)	4 (1.4)	87 (0.3)	.021
No (1.1)	287 (98.6)	24,391 (99.7)	

a Data are presented as n (%) unless otherwise indicated. 705 patients were excluded from the risk factor analysis due to not having surgery with hamstring or patella autograft. BMI, body mass index; VTE, venous thromboembolism.

**Table 2:** Perioperative Data of Patients With (n = 291) and Without (n = 25,018) Septic Arthritis^*a*^

	Septic Arthritis		
Variable (Incidence Rate)	Yes	No	*P* Value
Type of surgery (n = 25,309)			
Outpatient (1.2)	232 (79.7)	19,130 (76.5)	.192
Inpatient (1.0)	59 (20.3)	5888 (23.5)	
Primary (1.2)	276 (94.8)	23,622 (94.4)	.753
Revision (1.1)	15 (5.2)	1396 (5.6)	
Cartilage lesion (n = 25,309)			
Yes (1.4)	99 (34.0)	6780 (27.1)	.008
No (1.0)	192 (66.0)	18,238 (72.9)	
Meniscal suture (n = 25,309)			
Yes (1.2)	21 (7.2)	1668 (6.7)	.709
No (1.1)	270 (92.8)	23,350 (93.3)	
Choice of graft (n = 25,309)			
Hamstring tendon (1.2)	280 (96.2)	23,083 (92.3)	.012
Patellar tendon (0.6)	11 (3.8)	1935 (7.7)	
Operating time (n = 23,919)	82.0 (35-246)	74.5 (17-304)	<.001
<70 min (0.8)	84 (30.4)	10,631 (45.0)	<.001
≥70 min (1.5)	192 (69.6)	13,012 (55.0)	
Perioperative antibiotic (n = 24,744)			
Cloxacillin (1.1)	272 (94.8)	23,861 (97.6)	.002
Clindamycin (2.5)	15 (5.2)	596 (2.4)	
Perioperative antibiotic dose (n = 25,309)			
1 dose (1.2)	193 (66.3)	16,280 (65.1)	.657
≥2 doses (1.1)	98 (33.7)	8738 (34.9)	
Prolonged antibiotic prophylaxis (n = 25,309)			
Yes (0.7)	12 (4.1)	1638 (6.5)	.101
No (1.2)	279 (95.9)	23,392 (93.5)	

a Data are presented as n (%) or mean (range).

The differences from the univariate analysis (Tables 1 and 2) persisted, except for cartilage lesions, in the binary logistic regression analysis. Independent risk factors for SA were male sex (OR, 1.65; 95% CI, 1.28-2.13), operating time of ≥70 minutes (OR, 1.83; 95% CI, 1.42-2.36), use of hamstring tendon autograft (OR, 2.23; 95% CI, 1.21-4.08), and use of clindamycin (OR, 1.94; 95% CI, 1.10-3.41) (Table 3).

**Table 3:** Risk Factors for Septic Arthritis in a Logistic Regression Analysis^*a*^

	Reference Category	Beta Coefficient	OR	95% CI	*P* Value
Patient characteristics					
Male sex	Female sex	0.50	1.65	1.28-2.13	<.001
Perioperative data					
Cartilage lesion	No cartilage lesion	0.24	1.27	0.99-1.63	.062
Operating time ≥70 min	<70 min	0.61	1.83	1.42-2.36	<.001
Hamstring tendon autograft	Patellar tendon autograft	0.80	2.23	1.21-4.08	.010
Clindamycin	Cloxacillin	0.66	1.94	1.10-3.41	.022
Constant	N/A	−7.14	N/A	N/A	N/A

a N/A, not applicable; OR, odds ratio.

There were 4 patients with a VTE and SA. Three of the VTE events occurred between 8 and 33 days after the diagnosis of SA. One event of VTE occurred 11 days before the diagnosis of SA.

## Discussion

The incidence of SA after ACLR was 1.1% in this national cohort comprising 26,014 cases of ACLR, spanning the period between 2006 and 2013. Male sex, a longer operating time, the use of a hamstring tendon autograft, and the use of clindamycin compared with the use of cloxacillin were all independent risk factors for postoperative SA.

### Incidence

Our finding of an incidence of 1.1% SA is slightly higher than that of previous registry-based studies; however, the cohorts were substantially smaller than our cohort of 26,014 cases.^7,9,36^ Westermann and colleagues^36^ used the American College of Surgeons National Surgical Quality Improvement Program, a database including medical record information from 500 academic and private institutions in the United States. SA was defined by modified Centers for Disease Control and Prevention criteria. The incidence of SA was 18 of 6398 patients (0.28%). The Multicenter Orthopaedic Outcomes Network database was used by Brophy and coworkers.^7^ This database contains pre-, peri-, and postoperative patient information from 6 hospitals in the United States. Patients who had postoperative surgical irrigation and debridement were defined as having SA. No information on clinical data such as fever or a positive culture was available. Seventeen of 2198 patients (0.77%) were found to have SA.^7^ Cancienne and colleagues^9^ used the PearlDiver Patients Records Database, an insurance-based database of patient records, and compared tobacco users with a matched control group of nontobacco users. SA was defined as a combination of diagnosis and procedural codes, and 135 cases of SA were found among 13,358 patients (1.0%).

We defined SA by using a combination of national registries, including both diagnosis codes and prescribed antibiotics, and an analysis of medical records. Other advantages of our cohort included its high national coverage (>90%) and greater number of patients with SA (n = 298). One possible explanation for the different incidence rates of SA between the above-referred registry-based studies and ours could be a possible loss to follow-up. Our study design, with a combination of several methods, decreased the risk of loss to follow-up. When comparing our results with those of studies from single institutions, the incidence is more similar (0.58%-1.8%), which could be explained by a similarly low rate of loss to follow-up.^4-6,33^ Another possible explanation of the diverging incidence of SA is the distribution of graft choice, which has an effect on the incidence of SA. In our study population, the majority underwent surgery using hamstring tendon autograft (90.8%). The study by Brophy et al^7^ reported the graft choice, whereas the studies by Cancienne et al^9^ and Westermann et al^36^ did not provide such information.

SA after ACLR is thought to be caused by direct inoculation through the insertion of instruments and graft in the knee during surgery, whereas SA not related to surgery is thought to be caused by the hematogenous spread of bacteria.^27,32^ In the ACLR setting, the use of vancomycin to soak the graft has been reported to successfully reduce the rate of infection.^13,35^ To our knowledge, the use of vancomycin-soaked grafts in Sweden did not start during the study period. In a study of Swedish ACL surgeons performed 2017, 8% reported the use of vancomycin-soaked grafts.^12^

In 2013, Sechriest et al^28^ showed how the incidence of knee sepsis after ACLR can be extensively reduced by implementing a standardized treatment protocol that includes pre-, peri-, and postoperative care; at their institution, the incidence was lowered from 1.96% to 0%. The analysis of the 16 high-volume centers in our study revealed a wide spread of the incidence of postoperative SA (0.2%-2.9%), which could indicate differences in local routines. It is especially noteworthy that 1 center had only 2 infections (0.2%) during the study period, which demonstrates that a low incidence of SA is possible to achieve without using vancomycin-soaked grafts.

### Sex

Male sex increased the risk of SA and has not previously been proven to be a risk factor for SA after ACLR. Among patients with SA after invasive pneumococcal disease, male sex was an independent risk factor for SA.^23^ The same finding has been reported in studies analyzing risk factors for prosthetic knee joint infection, where male sex has been proven to be an independent risk factor for infection.^16,24^ Using the present data, we have not been able to explain whether this finding is because of the sex itself or is a proxy for risk factors not included in the analysis.

### Operating Time

An operating time ≥70 minutes was shown to be an independent risk factor for SA in our cohort, where patients with SA had a mean operating time of 82 minutes compared with 75 minutes among patients without infection. The association between a longer operating time and a higher risk of infection has been established in operative procedures in general.^11^ Agarwalla and colleagues^1^ used data from the American College of Surgeons National Surgical Quality Improvement Program database to show that an increase in operating time in ACLR increased the risk of surgical site infection. A study of the same database, but focusing on knee arthroscopic procedures excluding ACLR, found a linear relationship between an increase in operating time and an increase in surgical site infection and sepsis.^15^

### Graft Choice

The use of hamstring autografts compared with patellar tendon autografts increased the risk of SA (OR, 2.23). The same finding has previously been reported by several authors, and it is stipulated that the hamstring autograft is more easily contaminated during harvest and preparation.^3,7,21,25^ The hamstring tendon has a larger surface exposed to potential bacteria compared with a patellar or quadriceps tendon autograft, which could be one explanation of the increased risk of contamination. The majority of the ACLRs in our study were performed using a hamstring tendon autograft (90.8%), while only 7.6% were performed using a patellar tendon autograft. Different grafts have different properties, such as mechanical strength, harvest site morbidity, and rupture rates, and they are often chosen based on the surgeon’s preference, local routines, and patient characteristics.^14,20^ In Sweden, the use of hamstring autografts gained popularity at the beginning of the 2000s and peaked at 98% in 2012.^31^ Recent reports from the SKLR, however, have shown a slight decrease.^31^ The use of allografts in Sweden is very low, with only 142 cases (0.6%) during the study period and no report of infection. Allografts in Sweden are always presoaked in antibiotics, most often rifampicin, which potentially explains the low rate of SA. Yu et al^38^ reported 15 deep infections in a cohort with 10,190 allografts, corresponding to an incidence of 0.15%.

### Perioperative Antibiotic Prophylaxis

In our study, the use of clindamycin as perioperative antibiotic prophylaxis compared with cloxacillin was an independent risk factor that increased the risk of SA (OR, 1.94). This is a novel finding in the ACLR setting. Robertsson and colleagues^26^ reported similar results when analyzing the results after total knee arthroplasty, where clindamycin compared with cloxacillin increased the risk of revision due to infection by 50%. In Sweden, clindamycin is often used when the patient reports an allergy to penicillin. Robertsson and colleagues^26^ pointed out that a thorough review of the patient’s allergy history may reveal that it is not a case of type 1 allergy and that a second- or third-generation cephalosporin could safely be used instead.

### Diabetes Mellitus

Diabetes mellitus as a risk factor for postoperative SA has been discussed, and contradictory findings have been reported.^7,36^ In our population, 2 of 189 patients (1.1%) with diabetes mellitus had SA, and no significant correlation was found between postoperative infection and diabetes mellitus. Despite the fact that our cohort was large, the patients with diabetes mellitus only represented 0.7% of the total study population, and an analysis is therefore difficult to perform and is associated with bias.

### Outpatient Surgery

Westermann and colleagues^36^ showed that patients who are admitted to the hospital after an ACLR, compared with not being admitted, have a higher risk of postoperative SA. In our study, inpatient surgery was not associated with a higher risk of postoperative SA.

### Cartilage Lesions

In the univariate analysis, the SA group had a statistically significantly larger proportion of cartilage lesions (34.0%) compared with the control group (27.1%). The ACLRs with cartilage lesions had a longer operating time compared with ACLRs without cartilage lesions (79.3 vs 72.9 minutes), and it is therefore possible that the increase in SA is explained by the time effect. Another explanation could be that the cartilage lesion makes the knee more vulnerable to bacteria, which perhaps adhere more strongly to the damaged surface. However, the logistic regression analysis did not reveal a statistically significant relationship between SA and cartilage injury (OR, 1.27; 95% CI, 0.99-1.63).

### Prolonged Antibiotic Prophylaxis

Prolonged antibiotic prophylaxis was used in 1650 of all ACLRs (6.5%). In the SA group, it was used in 12 ACLRs (4.1%), but this was not statistically significant compared with the non-SA group. There is no support in the literature for prolonged antibiotic prophylaxis.^8^

### Smoking

In the ACLR setting, contradictory results regarding the effects of smoking and the risk of infection have been reported.^7,9^ In our study, smoking was not a risk factor for SA, and the total number of people who smoked was very low (5.9%); however, the variable is not complete because of a large number of missing values and should be interpreted carefully.

### Venous Thromboembolism

The incidence of VTE was significantly higher among patients with SA compared with patients without SA (1.4% vs 0.3%). However, 3 in 4 VTEs were diagnosed after SA. The causality between SA and VTE is probably inversed, with prolonged bed rest and immobility after SA increasing the risk of VTE.^2^

### Limitations

This study has several limitations. The incidence analysis was based on information from the NPR, which has a nationwide coverage >99%.^19^ However the positive predictive value of the NPR varies among different diagnoses (85%-95%).^19^ The specific ICD codes (M00 and T84) used in our study have not been analyzed. To summarize, this could have had an overall effect on the sensitivity of the study. In contrast, the specificity was increased by combining different methods in the selection process. The number of ACLRs with SA (n = 298) was small compared with that of the group without SA (n = 25,716), and several variables had small numbers, which could have affected the statistical analysis. Another limitation of our study is the lack of specific institutional data such as local routines in preoperative preparation of the patient, perioperative routines, and equipment in the operating room.

For future perspectives, our method of incidence analysis showed that using the Swedish registry information without a medical chart review yielded an approximately 5% (15 cases misclassified out of 315 cases that were checked) error rate for the detection of SA after ACLR.

## Conclusion

The incidence of SA after ACLR was 1.1%. Male sex, operating time ≥70 minutes, the use of a hamstring tendon autograft compared with a patellar tendon autograft, and the use of clindamycin as perioperative prophylaxis compared with cloxacillin were all independent risk factors for SA. Despite the fact that vancomycin to soak the graft was not used in this cohort, some units had an incidence of SA of 0.2%.

## Supplemental Material

sj-pdf-1-ajs-10.1177_0363546521993812 – Supplemental material for Risk Factors for Septic Arthritis After Anterior Cruciate Ligament Reconstruction: A Nationwide Analysis of 26,014 ACL Reconstructions Click here for additional data file. Supplemental material, sj-pdf-1-ajs-10.1177_0363546521993812 for Risk Factors for Septic Arthritis After Anterior Cruciate Ligament Reconstruction: A Nationwide Analysis of 26,014 ACL Reconstructions by Jesper Kraus Schmitz, Viktor Lindgren, Gunnar Edman, Per-Mats Janarv, Magnus Forssblad and Anders Stålman in The American Journal of Sports Medicine
