# Supplementary material for: Risk Factors for Septic Arthritis After Anterior Cruciate Ligament Reconstruction: A Nationwide Analysis of 26,014 ACL Reconstructions
Source: Am J Sports Med. 2021 Mar 25;49(7):1769–76. doi: 10.1177/0363546521993812 (PMC8182335; doi:10.1177/0363546521993812)
Supplement: sj-pdf-1-ajs-10.1177_0363546521993812 – Supplemental material for Risk Factors for Septic Arthritis After Anterior Cruciate Ligament Reconstruction: A Nationwide Analysis of 26,014 ACL Reconstructions [file sj-pdf-1-ajs-10.1177_0363546521993812.pdf]

Appendix Table A1. ATC code, type of antibiotic and dosage used in the selection process

ATC code	Antibiotic	Daily dose
J01CA04	Amoxicillin	750 mg 1x3
J01CR02	Amoxicillin and clavulanic acid	500/125 mg 1x3<br>875/125 mg 1x2
J01DB05	Cefadroxil	500 mg 1x2<br>1 g 1x2
J01MA02	Ciprofloxacin	250 mg 2x2<br>500 mg 1x2<br>750 mg 1x2
J01CE02	Phenoxymethylpenicillin	1 g 2x3
J01CF05	Flucloxacillin	500 mg 2x3<br>750 mg 2x3<br>1g 1x3
J01XC01	Fusidic acid	250 mg 2x3
J01FF01	Clindamycin	150 mg 1x3<br>300 mg 1x3
J01MA12	Levofloxacin	500 mg 1x1
J01XX08	Linezolid	600 mg 1x2
P01AB01	Metronidazole	400 mg 1x3
J01MA14	Moxifloxacin	400 mg 1x1
J04AB02	Rifampicin	600 mg 1x1
J01EE01	Sulfamethoxazole and trimethoprim	80/400 mg 2x2<br>160/800 mg 1x2
